# Opportunities and challenges for the clinical translation of structured DNA assemblies as gene therapeutic delivery and vaccine vectors

**DOI:** 10.1002/wnan.1657

**Published:** 2020-07-15

**Authors:** Marina A. Dobrovolskaia, Mark Bathe

**Affiliations:** ^1^ Nanotechnology Characterization Laboratory, Cancer Research Technology Program Frederick National Laboratory for Cancer Research sponsored by National Cancer Institute Frederick Maryland; ^2^ Department of Biological Engineering Massachusetts Institute of Technology Cambridge Massachusetts

**Keywords:** DNA origami, gene therapeutic, nonviral delivery vector, structured DNA assemblies, vaccine

## Abstract

Gene therapeutics including siRNAs, anti‐sense oligos, messenger RNAs, and CRISPR ribonucleoprotein complexes offer unmet potential to treat over 7,000 known genetic diseases, as well as cancer, through targeted in vivo modulation of aberrant gene expression and immune cell activation. Compared with viral vectors, nonviral delivery vectors offer controlled immunogenicity and low manufacturing cost, yet suffer from limitations in toxicity, targeting, and transduction efficiency. Structured DNA assemblies fabricated using the principle of scaffolded DNA origami offer a new nonviral delivery vector with intrinsic, yet controllable immunostimulatory properties and virus‐like spatial presentation of ligands and immunogens for cell‐specific targeting, activation, and control over intracellular trafficking, in addition to low manufacturing cost. However, the relative utilities and limitations of these vectors must clearly be demonstrated in preclinical studies for their clinical potential to be realized. Here, we review the major capabilities, opportunities, and challenges we foresee in translating these next‐generation delivery and vaccine vectors to the clinic.

This article is categorized under:

Therapeutic Approaches and Drug Discovery > Emerging Technologies

Biology‐Inspired Nanomaterials > Nucleic Acid‐Based Structures

Therapeutic Approaches and Drug Discovery > Nanomedicine for Oncologic Disease

## Graphical abstract

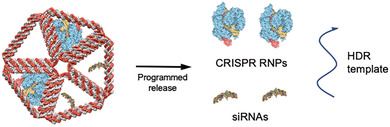

Virus‐like nucleic acid nanoparticles can be programmed to deliver and release CRISPR RNPs, HDR templates, and siRNAs either alone, or in combination, for next‐generation in vivo gene therapeutic applications.

## 1 INTRODUCTION

**FIGURE 1:**
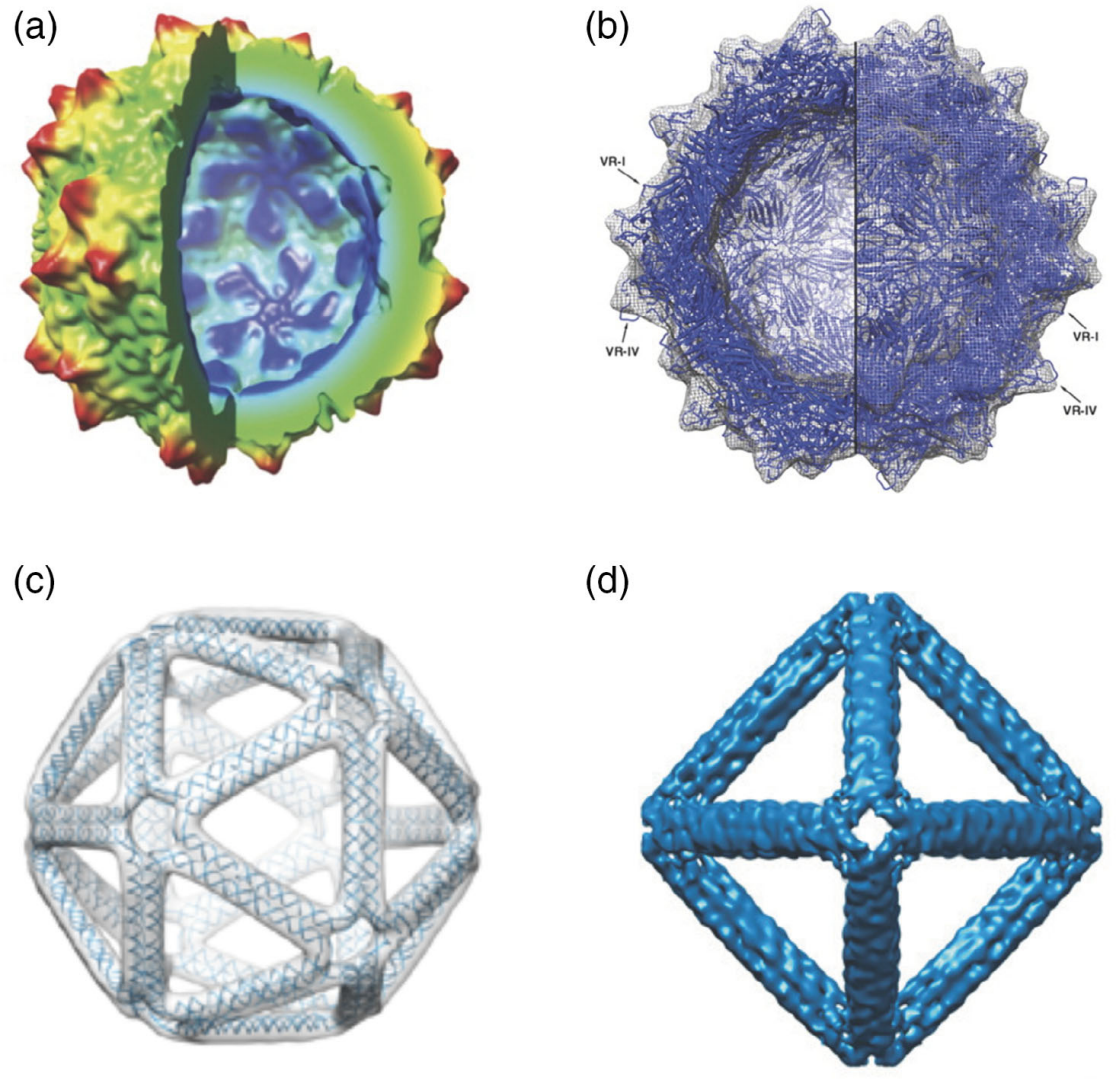
Structure‐composition comparison of AAV9 and virus‐like DNA assemblies fabricated using the scaffolded DNA origami approach. (a) Cryo‐EM density map of the icosahedral AAV9 capsid with an octant removed to show the capsid interior. The capsid consists of 60 self‐assembled copies total of viral proteins (VP) VP1, VP2, and VP3 in a ratio of 1:1:10, respectively. (b) Pseudoatomic model of AAV9 using AAV2 and AAV8 crystal structures as templates, showing the surface loops VR‐I and VR‐IV that control transduction phenotype and help determine tropism and antigenic reactivity in addition to transduction efficiency (DiMattia et al., 2012). The ssDNA AAV genome is approximately 4.8 kb and the AAV capsid is approximately 25 nm in diameter. (c) Cryo‐EM density map of an icosahedral DNA origami designed using two crosslinked duplexes per edge, where each edge is 52 basepairs or 18 nm long (Veneziano et al., 2016). The 3D atomic model generated by the automatic sequence design algorithm DAEDALUS is shown superposed on the EM density. The geometrically virus‐like particle is approximately 40 nm in diameter and contains a ssDNA scaffold of 3,120 bases. (d) Cryo‐EM density map of an octahedral DNA origami consisting of six crosslinked duplexes per edge, where each edge is 84 basepairs or 29 nm long (Jun, Shepherd, et al., 2019). The overall particle is approximately 41 nm in diameter and contains a ssDNA scaffold of 6,762 bases. Panels a and b are reproduced by permission from DiMattia et al. (2012). Panel c is reproduced by permission from Veneziano et al. (2016). Panel d is reproduced by permission from Jun, Shepherd, et al. (2019)

Gene therapeutic delivery vectors are typically classified into viral and nonviral, with the former principally including lentivirus and adeno‐associated virus (AAV) (Figure 1a, b), and the latter including cationic polysaccharides, synthetic polymers, lipids, and peptides. While major advantages of viral vectors include their organ‐ and cell‐specific targeting capabilities, together with their facile cellular entry and gene transduction efficiency, severe limitations include their manufacturing cost, immunogenicity, therapeutic payload versatility that is typically limited to ~4 kb trans‐genes for AAV, and lentiviral chromosomal integration that may induce long‐term genomic instability and cancer when used in vivo (Xiao, Shi, Qu, Chu, & Qian, 2019). In contrast, nonviral carriers have the ability to co‐formulate a diversity of gene therapeutic payloads including small interfering RNAs (siRNAs), anti‐sense oligos (ASOs), messenger RNAs (mRNAs), and CRISPR ribonucleoprotein complexes (RNPs), and they can have relatively low manufacturing costs and controlled immunogenicity, yet they often suffer from targeting limitations outside of the liver, low transduction efficiencies due to barriers associated with cellular entry and lysosomal escape, and often also are subject to in vivo instability due to nuclease degradation and renal or other clearance pathways (Yin et al., 2014).

## 2 VIRUS‐LIKE STRUCTURED DNA ASSEMBLIES

### 2.1 DNA origami formulation

**FIGURE 2:**
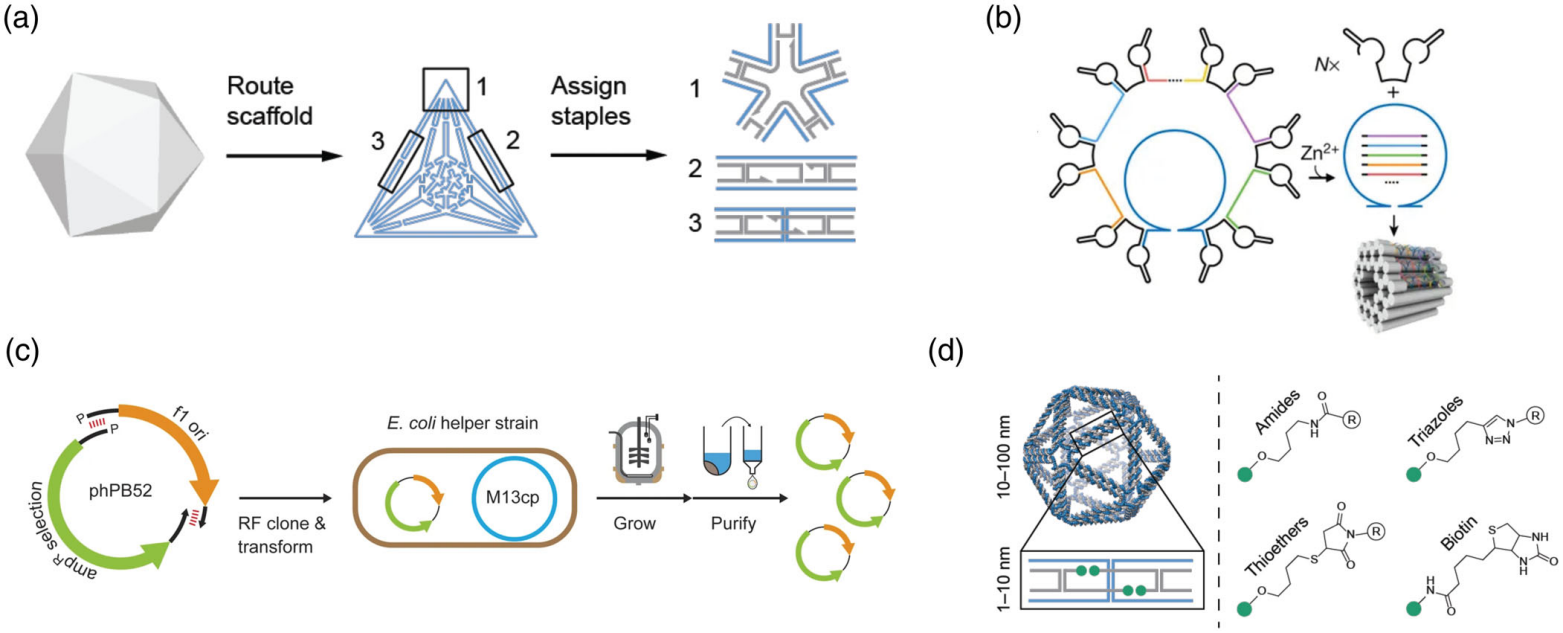
Formulation and synthesis of virus‐like structured DNA assemblies. (a) Fully automated design software DAEDALUS (Veneziano et al., 2016) for two‐helix bundle edges or TALOS (Jun, Shepherd, et al., 2019) for six‐helix bundle edges is used to route the ssDNA scaffold and complementary oligonucleotide staples that hybridize to the scaffold to self‐assemble the target virus‐like geometry. Any polyhedral geometry can be fabricated with edge lengths specified between ~10 and 50 nm. (b) Scalable biological production of custom sequence and length ssDNA staples can be achieved in *Escherichia coli* using an M13 phagemid and a helper phage or (c) ssDNA scaffold using a helper plasmid such as M13cp (Chasteen, Ayriss, Pavlik, & Bradbury, 2006) grown in a commercial bioreactor (Praetorius et al., 2017; Shepherd, Du, Huang, Wamhoff, & Bathe, 2019). Fixed scaffold sequences can be eliminated using either a split‐origin of replication strategy (Nafisi, Aksel, & Douglas, 2018) or self‐cleaving DNazyme inserts shown in (b) that result in linear instead of circular ssDNA (Engelhardt et al., 2019; Praetorius et al., 2017). Staples are shown in (b) as different colors in the circular ssDNA at left, with Zn^2+^ added to activate the DNazyme self‐cleaving reaction, which then results in the linear staples with small overhangs that then self‐assemble into the brick‐like origami shown. (d) Synthetic oligonucleotide staples vary in length between 20 and 60 nucleotides with free 3′ and 5′ ends that can be functionalized using a variety of chemistries (Wamhoff et al., 2019). Alternatively, they can be designed with ~10–20 nucleotide ssDNA overhangs of prescribed sequences that protrude either into or out of the particle, which complementary ssDNA, ssPNA, ssLNA, or ssRNA can then hybridize to for noncovalent, reversible attachment of either nucleic acid, small molecule, protein, or peptide targeting ligands, external to the particle, or therapeutic cargo, internal to the particle. Panel a: Reprinted with permission from Veneziano et al. (2016). Copyright 2016 The American Association for the Advancement of Science. Panel b: Reprinted with permission from Praetorius et al. (2017). Copyright 2017 Springer Nature. Panel c: Reprinted with permission from Shepherd et al. (2019). Copyright 2019 Springer Nature. Panel d: Reprinted with permission from Wamhoff et al. (2019). Copyright 2019 Annual Reviews, Inc.

Scaffolded DNA origami offers a nascent, alternative nonviral gene therapeutic delivery and vaccine platform with unique molecular characteristics and capabilities compared with its well‐established counterparts that include lipid nanoparticles, cationic natural and synthetic polymers, as well as antibodies and aptamers. The formulation was originally coined “DNA origami” by its inventor Paul Rothemund (2006) because of its fabrication approach, which consists of “folding” via thermal annealing the long, ~7 kb single‐stranded genomic DNA “scaffold” from M13 into structured two‐dimensional (2D) assemblies via Watson–Crick base‐paired hybridization to complementary synthetic oligonucleotides. Since this time, the approach has more broadly been termed “structural DNA nanotechnology” to reflect the highly organized, structured nature of the DNA assemblies that are the end‐product of interest for biomolecular applications (Bathe & Rothemund, 2017; Wamhoff et al., 2019). In the past decade, the technique has broadened considerably to synthesize both 2D and three‐dimensional (3D) structures on the 10–100 nanometer‐scale with exquisite geometric control, high structural fidelity, and near quantitative synthetic yield for certain classes of target shapes, principally of the wireframe variety (Wamhoff et al., 2019). This particularly versatile class of DNA origami is composed of virus‐like polyhedral structures in 3D (Jun et al., 2019; Veneziano et al., 2016) (Figure 1c, d) and mesh‐like structures in 2D (Jun et al., 2019; Jun, Wang, Bricker, & Bathe, 2019; Wamhoff et al., 2019). While wireframe geometries were originally conceived of by Yan and co‐workers using dual‐duplex edges for a few select geometries (Yan, Park, Finkelstein, Reif, & LaBean, 2003; Zhang et al., 2015), they were subsequently generalized by Bathe and co‐workers to 2D and 3D geometries using either two‐helix (Jun, Zhang, et al., 2019; Veneziano et al., 2016) or six‐helix bundle edges (Jun, Shepherd, et al., 2019; Jun, Wang, et al., 2019) for enhanced mechanical rigidity, and nearly any 2D or 3D shape, with synthetic DNA staple sequences programmed fully automatically by software based on simple target geometric representations (Jun, Shepherd, et al., 2019; Jun, Wang, et al., 2019; Jun, Zhang, et al., 2019; Veneziano et al., 2016; Wamhoff et al., 2019) (Figure 2a). An alternative strategy using single duplex wireframe edges offers semi‐automatic sequence design (Benson et al., 2015), yet with low shape fidelity and mechanical rigidity compared with two‐ and six‐helix bundle edge designs (Wamhoff et al., 2019).

Wireframe DNA origami now offers the ability to fabricate diverse classes of virus‐like geometries on the 10–100 nm scale with full control over internal therapeutic payload complexation that may include siRNAs (Hoiberg, Sparvath, Andersen, Kjems, & Andersen, 2019; Lee et al., 2012), ASOs, mRNAs, and CRISPR RNPs, as well as outward ligand and immunogen presentations that may include peptides, aptamers, lipids, sugars, small molecules, and proteins (Okholm & Kjems, 2016; Veneziano et al., 2020; Wamhoff et al., 2019). Similar to their applications in RNA nanotechnology (Panigaj et al., 2019), aptamers are anticipated to facilitate active targeting of DNA origami to specific cellular subtypes, tissues, and possibly even organs, with the capability of conditional payload release (Douglas, Bachelet, & Church, 2012). Thus, reproducible, high‐throughput, and cost‐effective synthetic strategies at preclinical scale together with in vivo and in vitro characterization of stability, immunogenicity, toxicity, targeting efficiency, and gene or immune cell modulation are the current foci of numerous translational preclinical research programs internationally.

Synthetic strategies for the fabrication of scaffolded DNA origami objects has traditionally relied on using the ~7 kb circular single‐stranded DNA genome from M13 produced either in large‐scale bioreactors commercially available from providers such as NEB, Inc., or in‐house in academic labs and facilities. Recently, enzymatic (Ducani, Kaul, Moche, Shih, & Högberg, 2013; Krieg & Shih, 2018; Veneziano et al., 2016, 2018) and M13 phage based production systems (Chasteen et al., 2006; Ducani et al., 2013; Engelhardt et al., 2019; Nafisi et al., 2018; Praetorius et al., 2017; Shepherd et al., 2019) have been used to engineer sequence control below the ~15 and 10kb limits respectively imposed by polymerase chain reaction based amplification and M13. Bacterial production offers staple sequence control using the strategies of Ducani et al. (2013) and Praetorius et al. (2017), who respectively introduced restriction enzyme and DNAzyme cutting sites to produce staples from helper phage genomes (Figure 2b). Sequence‐controlled scaffolds can also be produced bacterially using the approach of Nafisi et al. (2018), who implemented a split‐origin of replication strategy to eliminate all but ~300 nucleotides of fixed sequence from M13, or Engelhardt et al., (2019) who introduced DNAzyme cutting sites to produce linear scaffolds with minimal fixed sequence overhangs. Like Nafisi et al. (2018), Shepherd et al. (2019) also utilized the M13cp helper plasmid from Chasteen et al. (2006), and used batch fermentation to demonstrate scalable ssDNA production without dsDNA contamination (Figure 2c). The preceding approaches can now be used to eliminate any protein‐coding or promoter regions in ssDNA scaffolds used in therapeutic applications, and also offer the capability to produce custom sequence and length single‐stranded DNA for use as CRISPR homology directed repair (HDR) templates (Cong et al., 2013; Lee et al., 2017; Lin, Staahl, Alla, & Doudna, 2014), or to control numbers of CpG domains present for immune stimulation through TLR9 sensing pathways (Ablasser & Chen, 2019; Civril et al., 2013; Comberlato, Paloja, & Bastings, 2019; Gluck & Ablasser, 2019). While bacterial production offers near‐linear scalability in cost of scaffold production in bioreactors that may reach 1,000–10,000 L and larger‐scales for clinical studies, enzymatic synthesis approaches offer full control over scaffold sequence and length, as well as nucleotide analog composition for modulation of immunostimulatory or serum stability properties. However, large‐scale enzymatic production requires industrial‐scale thermal cycling using continuous flow batch production or a related approach (Kopp, de Mello, & Manz, 1998). Future biological production strategies might incorporate nucleotide analogs within bacterial expression systems to control immunostimulatory and stability properties of ssDNA scaffolds, akin to synthetic therapeutic mRNA production (Cao et al., 2019; Kariko, Muramatsu, Ludwig, & Weissman, 2011; Svitkin et al., 2017), and enzymatic approaches might leverage isothermal synthesis to facilitate scalable production.

In contrast to the long ssDNA scaffold used to structure DNA assemblies using DNA origami, oligonucleotide staple strands required to hybridize the DNA scaffold to promote self‐assembly through thermal annealing are typically produced using standard, solid‐phase phosphoramidite synthesis, akin to the production of therapeutic siRNAs and ASOs, with full control over backbone and nucleotide modifications to control immunomodulation and serum stability (Chernikov, Vlassov, & Chernolovskaya, 2019; Ozcan, Ozpolat, Coleman, Sood, & Lopez‐Berestein, 2015; Shen & Corey, 2018; Wu et al., 2014) (Figure 2d). Unmodified oligonucleotide staple strands may alternatively be produced biologically at scale using phage based single‐stranded DNA production followed by enzymatic cleavage (Praetorius et al., 2017), or enzymatically using rolling circle amplification (Ducani, Nature Methods, 2013; Schmidt et al., 2015).

Several unique attributes of wireframe DNA origami consisting of dual‐duplex edges includes their structural stability in physiological salt concentrations (Veneziano et al., 2016) compared with traditional, brick‐like origami that require upwards of 10–14 mM MgCl_2_ to remain folded without crosslinking (Gerling, Kube, Kick, & Dietz, 2018) or cationic polymer‐induced stability (Ponnuswamy et al., 2017). The relatively low molecular weight of wireframe objects also reduces the amount of DNA required for delivery, which may benefit cellular uptake and toxicity. However, their polyhedral edges that consist only of two crosslinked duplexes each may be more susceptible to enzymatic degradation in vivo, primarily through the action of DNase‐1, a nonspecific minor‐groove binding endonuclease that acts nonspecifically to digest chemically unprotected or unmodified DNA origami objects (Castro et al., 2011; Conway, McLaughlin, Castor, & Sleiman, 2013; Ponnuswamy et al., 2017), which clearly needs to be addressed through lipid encapsulation (Perrault & Shih, 2014; Yin, Kauffman, & Anderson, 2017) or other protective strategy if the ssDNA scaffold were to be used as an HDR template. The preceding stabilization strategies for brick‐like origami may also be used to protect DNA‐based assemblies from DNase‐1 activity that typically results in degradation in serum and in vivo within tens of minutes to hours (Jiang et al., 2015; Okholm et al., 2014; Perrault & Shih, 2014; Ponnuswamy et al., 2017; Raniolo et al., 2019).

While several of the preceding approaches have demonstrated increased stability in vitro and in vivo, considerable room is still open for testing alternative natural and nonnatural stabilization strategies ranging from cationic polysaccharides and polymers to minor‐groove‐binding agents, and backbone or nucleotide modifications. In each case, minimal modifications should be implemented as required by the specific in vivo application of interest in order to minimize introduction of manufacturing complexities, costs, and possible off‐target toxicity. In addition, gene therapeutic applications may benefit from leveraging the natural biodegradability of DNA origami in vivo that may in fact be used to program temporal and spatial scaffold degradation for therapeutic siRNA, ASO, or other targeted therapeutic payload release, either through the passive action of extra‐cellular DNase‐1, or intracellular, lysosomal DNase‐2, or alternatively through pH‐sensing and response within tumors and endosomal trafficking pathways.

### 2.2 Gene therapeutic and vaccine delivery

DNA origami formulations have several unique characteristics as delivery or vaccine vectors including their: (a) homogeneity in chemical composition; (b) customizable 10–100 nm size; (c) virus‐like geometry to spatially template peptides, aptamers, sugars, lipids, and other small molecules or antigens for receptor‐mediated cellular targeting and uptake through controlled avidity (ligand copy numbers presented) and specificity (multiple ligand types); (d) capability to be co‐formulated with siRNAs, ASOs, mRNAs, or CRISPR‐RNPs; (e) nucleic acid based composition for triggering endosomal TLR9 and cytosolic DNA sensors for controlled immunostimulation; and (f) their synthetic composition that offers full control over their cGMP production and characterization, at low cost compared with biologics such as viral vectors that can reach costs of $1m per patient for high‐dose AAV therapeutic treatments such as Duchenne Muscular Dystrophy. One successful application of DNA origami as an in vivo vaccine was demonstrated by the team led by Y. Chang; tetrahedron DNA origami containing a CpG oligo as an adjuvant were used to deliver protein antigen and resulted in a robust antibody response and the formation of immunological memory against the antigen (Liu, 2012; Liu et al., 2012). More recently, Veneziano et al. (2020) used DNA origami to elucidate in vitro the relative roles of antigen copy number, spacing, and affinity on B cell activation using the clinical HIV‐1 vaccine candidate eODGT8 (Jardine et al., 2013, 2015). They found maximal B cell activation was achieved when antigens were spaced ~25–30 nm apart, and that as few as 5 of these antigens could activate B cells to similar levels as a 60‐mer protein nanoparticle, which will be interesting to test in vivo (Jardine et al., 2016; Sok et al., 2016). Importantly, the facile HIV‐1 antigen conjugation strategy to DNA origami employed in this work is easily transferable to other viral pathogens such as SARS‐CoV‐2 (Walls et al., 2020; Yan et al., 2020; Yuan et al., 2020), as well as to B cell and T cell peptide epitopes for a variety of therapeutic applications (Heesters, van der Poel, Das, & Carroll, 2016; Irvine, Hanson, Rakhra, & Tokatlian, 2015).

**FIGURE 3:**
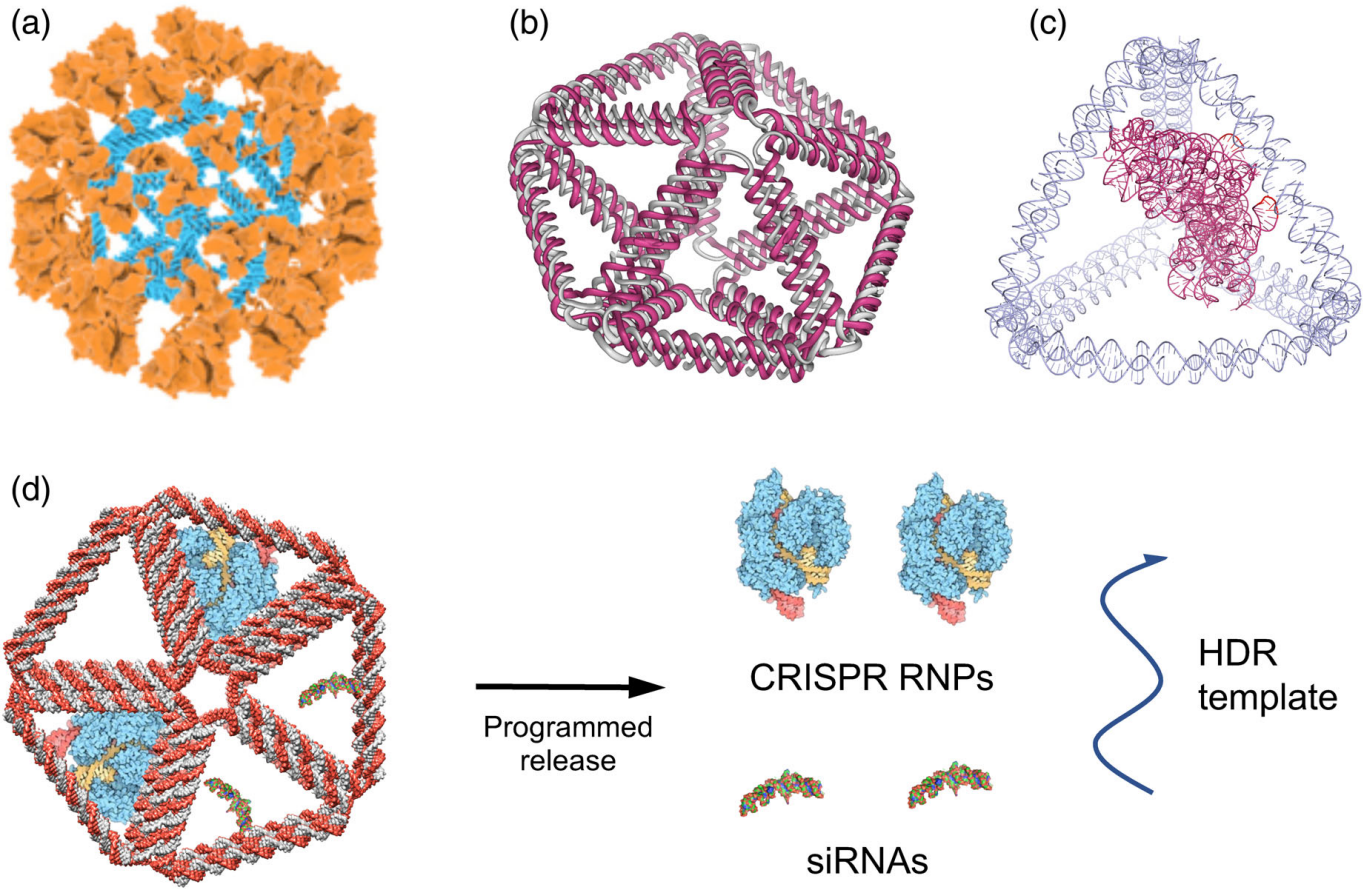
Therapeutic modalities with structured DNA assemblies. (a) Any virus‐like structured DNA assembly can be used to present ligands outwardly for immune cell stimulation, cell targeting, intracellular trafficking, or some combination of these. Thirty copies of the HIV envelope glycoprotein gp120 are shown conjugated to the outside of the icosahedral DNA origami with two‐helix bundle edges. (b) Instead of using a ssDNA scaffold to fold virus‐like DNA nanoparticles, messenger RNA scaffold can be used, wherein the structure shows the ~1 kb nucleotide mRNA from Green Fluorescent Protein folded into a pentagonal bipyramid by designing complementary ssDNA staples (Parsons et al., 2019). Staples could be functionalized with 3′ and 5′ targeting or other moieties, as in (a). (c) Alternatively, the mRNA, in this case RFP, can be co‐formulated to hybridize to the interior of the fully DNA origami nanoparticle, in this case a simple tetrahedron with two‐helix bundle edges, by presenting inward facing ssDNA overhangs that are complementary to loop regions of the mRNA (Bathe lab, unpublished data). (d) Similarly, CRISPR RNPs can be co‐formulated with ssDNA HDR template by hybridizing the guide RNAs to complementary ssDNA overhangs on the interior of the pentagonal bipyramid structured DNA assembly shown, with multiple siRNAs, all of which can in principle be released in a programmed manner using pH‐ or receptor‐mediated triggering mechanisms

Additional attractive features of DNA‐ and RNA‐based structural assemblies formed using principles of DNA origami fabrication include the ability to incorporate logic‐based sensing and switches for controlled disassembly or payload release in response to tumor or endosomal pH, target receptor‐binding, or controlled intracellular trafficking pathways including retro‐grade trafficking to facilitate cytosolic entry, and the possibility to exploit 3D size, geometry, and spatial antigen presentation to control alternative endocytic intracellular uptake pathways in the modulation of gene expression and immune cell activation (Kumari, Swetha, & Mayor, 2010; Mayor & Pagano, 2007; Mosesson, Mills, & Yarden, 2008). And while ASOs, siRNAs, mRNAs, and CRISPR RNPs can easily be co‐formulated with structured DNA assemblies using templated hybridization or covalent conjugation, chemical or other stabilization strategies together with controlled release are in principle needed to attain therapeutic cellular targeting and action (Figure 3).

Notwithstanding, the historical development of FDA‐approved siRNA and ASO therapies have instructed that the simplest gene therapeutic constructs with appropriate in vivo efficacy are the most viable for clinical treatments, thereby minimizing complications due to manufacturing and/or toxicity and immunogenicity. This experience also teaches us to focus on the unique translational impact of therapeutic efficacy or index when using structured DNA assemblies as gene therapeutics or vaccines, rather than focus excessively on fundamental biological questions of stability, endosomal escape, and toxicity in model systems, which is of utmost importance to bear in mind during key preclinical validation stages for expedient clinical translation.

## 3 TRANSLATIONAL QUESTIONS

Several parameters determine what makes a new chemical or biological entity a drug candidate. These include potency, stability in a biological matrix, specificity to the target of interest, safety, pharmacokinetics (PK), pharmacodynamics (PD), and clearance profiles favorable to the intended indications (Kornbrust et al., 2013). Drug products are separated into three large categories—small molecules, biologics, and therapeutic nucleic acids (TNAs)—according to their structure; molecular weight; off‐target effects; absorption, distribution, metabolism, and excretion (ADME); PK; and immunogenicity (Kornbrust et al., 2013). TNAs are diverse and include both short oligonucleotides such as ASOs and siRNAs and larger nucleic acids such as mRNAs. Translation of these concepts from bed to bedside uncovered some similarities between TNAs, small molecules, and biologics, as well as several unique properties that resulted in TNAs being categorized as a class separate from biologics and small molecules. More recently, self‐assembling nucleic acid nanoparticles (NANPs) made of DNA and RNA oligonucleotides were separated from the TNA category due to several unique aspects concerning their molecular weight, size, off‐target toxicity, and manufacturing (Dobrovolskaia, 2016). To date, however, it has not yet been formally determined whether structured DNA assemblies fabricated using the principle of scaffolded DNA origami belongs to the same category as NANPs, or deserves yet another separate category of drug products.

Previous studies have described the challenges commonly experienced during the bench‐to‐bedside translation of traditional TNAs, as well as the gaps in the current translation of NANPs (Dobrovolskaia, 2016; Dobrovolskaia & McNeil, 2015a; Henry, Geary, Yu, & Levin, 2001; Levin, 1999). Here, we focus on the current state‐of‐the‐art, translational challenges, and knowledge gaps pertaining specifically to DNA origami assemblies. Many questions relevant to the translation of DNA origami and corresponding knowledge gaps overlap with those pertinent to NANPs. Top‐level challenges are summarized in Table 1, and are reviewed further below.

**TABLE 1:** Key challenge areas in the translation of DNA origami to therapeutics

Area or parameter	Importance
Manufacturing	Clinical trials require large quantities of materials. Compatibility with scale‐up is a common bottleneck for new drug candidates. The ease of production may tremendously influence the cost
Analytical and bioanalytical methods	Detailed, transferable protocols for physicochemical characterization; detection of endotoxin and other IIMIs; and sterility, PK/PD, and immunogenicity‐enabling studies are needed to support IND and prepare CMC and investigator brochures
Formulation	Influences shelf‐life stability, compatibility with the intended route of administration, delivery (if needed), safety profile, cost, and compatibility with scale‐up
Distribution and accumulation in target tissues	Influence the selection of indication, prediction, and overcoming of toxicity
Circulation time and clearance	Contribute to both safety and efficacy. Ephemeral circulation is usually associated with a lack of efficacy, while prolonged circulation creates long‐term safety concerns
Local concentration at the injection site and a potential for systemic exposure	Determine the route of administration, influence safety, and contribute to the design of studies required for regulatory approval
Relationship between distribution to tissues and kinetics in plasma	Necessary for the dose selection and adjustment; must be considered in the context of a carrier used to improve tissue penetration
Protein binding and distribution to MPS	Control clearance rates, contribute to toxicity and influence bioavailability and efficacy
Mechanism of metabolism and metabolic profile	Contribute to safety and efficacy
Distribution into and inside cells	Necessary to design targeting and delivery strategies to achieve a desirable therapeutic effect and avoid side effects
Safety	Distribution to off‐target cells and tissues creates a potential for off‐target toxicity. If DNA origami is used as a delivery platform, it may change the distribution of a drug and create toxicity in tissues (e.g., kidney or MPS) where the drug would not distribute otherwise. Autoimmunity (breaking tolerance to DNA), cytokine response due to the recognition by TLRs and other innate immune receptors, hypersensitivity reactions due to the complement activation, and potential of hemorrhage due to the inhibition of the plasma coagulation cascade are the primary immunological safety questions that have not been adequately addressed. Toxicity to the kidney should be considered since the kidney is the primary organ of the accumulation of these particles. Genotoxicity and reproductive and developmental toxicity also require assessment; however, the information is not currently available

*Note*: The key areas, their importance, and current state are summarized.

Abbreviations: CMC, chemistry, manufacturing, and controls; IIMI, innate‐immunity‐modulating impurity; IND, investigational new drug application.

### 3.1 Compatibility with scale‐up and manufacturing costs

Both compatibility with scale‐up and manufacturing costs represent common challenges for many new technologies' successful clinical translation. A successful synthetic procedure reproducibly generates a nanoparticle product with desirable properties (e.g., size, targeting, drug loading). The issue experienced by many nanomedicine manufacturers is that, unlike small molecules, linear scale‐up of individual components rarely generates a homogenous nanoparticle product with the required physicochemical and functional attributes (Desai, 2012). Additional challenges include the final nanomedicine product costing an order of magnitude higher than legacy drugs, a result of complicated manufacturing, characterization, and regulatory approval processes (Goldberg et al., 2013). Sterilization represents a special challenge for nanomedicine manufacture, as many nanomaterials do not tolerate traditional methods for the terminal sterilization of a final drug product. As such, many nanomedicines require aseptic conditions, which raises the manufacturing cost (Desai, 2012).

Although the cost of DNA origami production varies depending on the scale and method of manufacturing, it is within the average range for other nanotechnology platforms commonly used in drug delivery (Table 2). The current cost estimates of DNA origami production are encouraging; however, it is not unreasonable to expect that good manufacturing practice (GMP) manufacturing under aseptic conditions and purification of DNA origami from endotoxin may substantially raise the cost of a drug product based on this technology, depending on the specific co‐formulation of gene therapeutics or small molecules and targeting ligands.

**TABLE 2:** Cost of DNA origami production in comparison to other nanotechnology platforms

Technology	Type of manufacturing and scale, if available	Cost per milligram, USD
DNA origami	Reaction systems shake flask (6‐L scale)	141.59
	Stirred tank (1.8‐L scale)	25.98
	Stirred tank reactor (800 L)	0.20
PEGylated liposomes	Nanosizer extrusion	141.50
PAMAM G7 dendrimers	N.A.	2
Gold colloids	N.A.	47–215
Iron oxides	N.A.	18.20

*Note*: Clinical trials require large amounts of a drug product. Manufacturing costs and technology compatibility with scale‐up are often a bottleneck to nanomedicine's translation from bench to bedside. In the field of nanotechnology‐formulated drugs, the production of a formulation equivalent to that of at least 1 kg of API is a typical prerequisite for a successful clinical program. The costs summarized in the table compare DNA origami with some of the most common nanotechnology platforms and are based on references (Ebiohippo, n.d.; Halford, 2005; Jasinski, Binzel, & Guo, 2019;Nanocomposix, n.d.; Praetorius et al., 2017; TTScientific, n.d.; US‐Nano, n.d.). The costs were converted to U.S. dollars (USD) based on the rates available on April 12, 2019. The costs reflect non‐good‐manufacturing‐practice (GMP) manufacturing of an unfunctionalized platform; the actual cost of manufacturing GMP‐grade nanotechnology drug product could be substantially different.

Abbreviation: N.A., not available.

### 3.2 Distribution to and accumulation in tissues of interest

Distribution and accumulation in tissues of interest, PK and PD, are the essential criteria typically used in the pharmaceutical industry for the selection of a drug's indication. Although TNAs are used for a variety of indications, only a handful of these concepts reached the approval stage (Levin, 2017, 2019; Oberemok et al., 2018). The main indication extensively explored for NANPs is cancer (Etheridge et al., 2013), while emerging applications include vaccines and immunotherapies (Chandler & Afonin, 2019). Similarly, DNA origami are also being considered for cancer and vaccine indications (Liu et al., 2012; Zhang et al., 2014). As of now, neither NANPs nor DNA origami technologies have received U.S. Food and Drug Administration approval for clinical use; both are still undergoing discovery and early preclinical development.

A desirable intracellular distribution profile and efficient uptake by target tissues and cell types are critical for the success of a new drug candidate. Traditional TNAs typically distribute to the skin, liver, eyes, and kidney (Geary, Leeds, Henry, Monteith, & Levin, 1997; Henry, Monteith, & Levin, 1997; Kornbrust et al., 2013; Levin, 1999, 2017, 2019). As such, they are commonly directed against targets in these tissues. A recent report demonstrated that, regardless of shape (rectangular, triangular, or tubular), DNA origami preferentially accumulated in the kidney within the first 12 hr postinjection (Jiang et al., 2018). Not surprisingly, similar to TNAs, DNA origami was proposed as drug candidates against targets in the kidney (Jiang et al., 2018).

In contrast, another study reported that tubular and square origami preferentially accumulated in the kidney and liver, whereas their triangular counterparts did so mainly in tumor tissue (Zhang et al., 2014). This difference in tissue accumulation profile between origami with different geometric shapes and sizes described in these studies (Jiang et al., 2018; Zhang et al., 2014) suggests that the presence of a tumor may influence DNA origami's tissue distribution. Therefore, selecting and testing indications based on the tissue distribution requires consideration of both the particles' geometric shape and size and the animal/disease model. The type of tumor model may play a major role in observed biodistribution, and needs to be presented and considered carefully in any investigative study, for example ectopic versus orthotopic, in order to avoid making too general conclusions at this early stage of development, as discussed elsewhere (Gengenbacher, Singhal, & Augustin, 2017). Further, given enzymatic degradation pathways in vivo through DNase action, while challenging, detailed in situ characterization of 2D/3D structure and composition of DNA origami is of immense importance to understand rigorously the mechanism of trafficking and delivery based on theoretical or in vitro models (Lacroix, Vengut‐Climent, de Rochambeau, & Sleiman, 2019; Okholm et al., 2014).

### 3.3 Circulation time and clearance

Circulation time and clearance rate are important criteria for ensuring a drug candidate's desirable efficacy. For this reason, a variety of nanotechnology carriers have found a unique niche in drug delivery by extending the circulation time of small molecules and biologics and allowing their accumulation in target tissues (Stern et al., 2010; Yoo, Chambers, & Mitragotri, 2010). A minimum circulation time of 6 hr is commonly used in nanotechnology as an indicator of a successful carrier (Stern et al., 2010; Yoo et al., 2010). Most nanotechnology concepts approved for clinical use have a circulation time between 6 and 24 hr (Stern et al., 2010; Yoo et al., 2010). In contrast, retention in the body for a prolonged period is commonly viewed as a safety concern, especially due to a potential of long‐term toxicity (Tyner et al., 2017; Weaver et al., 2017). Nonbiodegradable nanocarriers such as gold colloids, iron oxides, and mesoporous silica, share a common limitation in that they accumulate in the body over the time required for the drug to perform its therapeutic function (Tyner et al., 2017; Weaver et al., 2017). In this respect, DNA origami appear to be a promising platform because they start accumulating in the target tissue as early as 3 hr and are completely cleared from the body within 24 hr (Jiang et al., 2018). However, rigorous in vivo characterization of DNA origami stability and degradation timelines is still required, depending on the specific route of administration (e.g., intramuscular versus intravenous) and structured DNA assembly‐drug formulation considered.

### 3.4 Biodistribution

The local concentration of nanoparticles at the injection point and distribution to the systemic circulation following nonintravenous administration represent the next important translational parameters, which in turn depend on particle stability.

DNA molecules with a natural phosphodiester backbone are generally more stable chemically than RNA (Oberemok et al., 2018). This stability can be increased further by a variety of chemical alterations including, but not limited to, phosphodiester and methylphosphonate modifications of phosphate groups, 2′‐fluoro modification of the sugar moiety, and morpholino backbones that stabilize against nucleases (Oberemok et al., 2018). When DNA is organized into complex 3D origami structures, nuclease and ionic stability also depend on the number of duplexes crosslinked per edge (e.g., 2‐ versus 6‐ versus 24‐helix bundles) and the presence of magnesium and sodium ions (Kielar et al., 2018), as noted at the outset of this review. This creates two translational questions: first, regarding formulation shelf‐life, and second, concerning the stability of these structures upon dilution into injection buffers (e.g., saline) before administration, into biological matrices in vivo after administration, or both. As noted earlier, the conditions typically used to assemble two‐helix wireframe DNA origami allow reasonable shelf life in PBS, whereas wireframe or brick‐like assemblies composed of larger numbers of duplexes may require internal crosslinking or additional stabilization with cationic polymers because the 10–14 mM concentrations of magnesium needed for stability of the latter constructs are unphysiological. Because decreases in the concentration of magnesium ions to physiologically relevant levels will denature some DNA origami structures more than others, irrespective of crosslinking and cationic polymer‐induced stabilization, this will lead to the formation of imperfect structures and raise questions about toxicity and off‐target effects of both the parent DNA origami and these derivatives. Interestingly, low, physiologically relevant levels of magnesium ions in cell culture medium were observed in one study to affect brick‐like 3D DNA origami (octahedron and 24‐helix nanorod), but not 6‐helix‐bundle structures (Hahn, Wickham, Shih, & Perrault, 2014), which may be used to program diverse virus‐like DNA assemblies as delivery and vaccine vectors (Jun, Shepherd, et al., 2019). Importantly, irrespective of the type of stabilization implemented, it is currently incompletely understood how long DNA origami (unformulated, complexed with a protective coating, or encapsulated into a carrier) remains at the site of injection, whether it distributes systemically, and, if so, at what dose levels. Since magnesium ion levels differ between tissues (Jahnen‐Dechent & Ketteler, 2012), it would be interesting to understand whether or not origami stability and retention may also vary in different tissues. Studies answering these questions represent important milestones for the translation of these formulations to the clinic.

The data regarding the relationship between the total administered dose of the DNA origami, the dose delivered to the organ of interest and concentration within target cells, and their pharmacologic activity are needed to understand the kinetics of DNA origami and their therapeutic cargos at the site of action. To our knowledge, there are currently no available studies evaluating these parameters for DNA origami of various sizes and geometric shapes, where the former may be expected to impact overall biodistribution to organs and tissues, whereas the latter may be anticipated to strongly affect cellular uptake and trafficking pathways, particularly when combined with multi‐valent ligand presentations.

### 3.5 Protein corona, MPS clearance, and intracellular distribution

According to our current understanding of nanoparticle PK, systemically administered nanomedicines are distributed and cleared based on two main mechanisms: enhanced permeability in retention (limited to cancer‐bearing models) and the mononuclear phagocyte system (MPS) (Stern, Martinez, & Stevens, 2016). Plasma proteins' binding to nanoparticles depends on particle size and surface properties and has dramatic consequences for particle circulation times and clearance by the MPS (Bertoli et al., 2014; Bertoli, Garry, Monopoli, Salvati, & Dawson, 2016; Deville et al., 2016; Gibbons et al., 2015; Kelly et al., 2015; Maiolo, Bergese, Mahon, Dawson, & Monopoli, 2014; O'Connell et al., 2015; Wan et al., 2015; Wang et al., 2013). The so‐called “protein corona” was also shown to influence nanomedicines' targeting properties and affect particle distribution inside the cells (Bertoli et al., 2016; Mahon, Salvati, Baldelli Bombelli, Lynch, & Dawson, 2012; Wang et al., 2013). The majority of studies investigating the relationship between nanoparticles, protein corona, and particle distribution to and inside of the cells used “hard” nanomaterials, such as colloidal metals, metal oxides, and silica (Bertoli et al., 2014, 2016; Deville et al., 2016; Dobrovolskaia et al., 2014; Gibbons et al., 2015; Kelly et al., 2015; Maiolo et al., 2014; O'Connell et al., 2015; Wan et al., 2015; Wang et al., 2013). Studies of “soft” materials such as liposomes and dendrimers are limited to total protein binding due to the nuances in methodologies currently available for separating nanoparticle‐bound proteins from the bulk plasma (Ilinskaya & Dobrovolskaia, 2016).

Although it is logical to expect that proteins that function by binding to DNA would also bind to DNA origami, it is hard to predict the binding of other intracellular and plasma proteins to origami. Research to date has focused on designing DNA origami either to specifically bind proteins (e.g., growth factors, restriction enzymes, pathogen biomarkers, and thrombin) (Godonoga et al., 2016; Ke, Meyer, Shih, & Bellot, 2016; Koirala et al., 2014; Rinker, Ke, Liu, Chhabra, & Yan, 2008) or to encapsulate and deliver them (e.g., therapeutic antibody) (Douglas et al., 2012). DNA origami were also investigated as a cargo of protein‐based particles (e.g., viral‐capsid‐protein‐based carriers) (Sprengel et al., 2017) and as a way to arrange proteins into patterns with defined shapes (Busuttil, Rotaru, Dong, Besenbacher, & Gothelf, 2013).

The mechanisms of DNA origami uptake and intracellular distribution, along with the dependence of these parameters on physicochemical properties, method of synthesis, delivery vehicle (if applicable), and purity, require thorough investigation before clinical studies begin. To our knowledge, however, results from such studies are not yet available publicly.

### 3.6 Mechanism of metabolism and metabolic profiles

Since DNase‐1 is most common in plasma (Kishi et al., 1990), it influences the stability, metabolism, and clearance of DNA origami, as noted earlier. Moreover, several isoforms of DNase‐1 have been reported, and they vary between different tissues in the same individual, between individuals, as well as between species (Kishi et al., 1990). Therefore, one could also expect various rates of metabolism of DNA origami between various individuals and between plasma, the kidney, the liver, and—perhaps—other tissues. Differences are also possible in the metabolism and clearance between species commonly used in preclinical research (e.g., mouse, rat, dog, and monkey). Currently, it is unknown if the molecular weight, geometric shape, size, and chemical modification(s) of the scaffold and staples of DNA origami influence their metabolism. It would also be interesting to see whether DNA origami's metabolic profile and kinetics are similar to that of antisense DNA oligonucleotides that have already been investigated in both preclinical and clinical studies (Geary, Baker, & Crooke, 2015; Graham et al., 1998; Santos et al., 2015). Therefore, detailed preclinical animal studies in various species and a clinical study are required to improve our current knowledge base about DNA origami. Experimental challenges associated with metabolism studies of antisense DNA oligonucleotides have been reported (Ewles, Goodwin, Schneider, & Rothhammer‐Hampl, 2014), and could be used to guide similar studies of DNA origami.

### 3.7 General and class‐related toxicity

Sequence and shape‐dependent and ‐independent toxicities, especially to the kidney and MPS (organs identified as primary for the accumulation and clearance of DNA origami), are incompletely understood. Dose range‐finding studies and assessments of the maximum tolerated dose at the clinically relevant route of administration in rodent and nonrodent species are needed to determine DNA origami's general and class‐related toxicities. Immunotoxicity, especially immunogenicity; the potential to break tolerance to self‐DNA (autoimmunity); and acute toxicity related to effects on blood coagulation cascade, cytokines, interferon response, and complement activation represent a special safety focus area and are reviewed in more detail below. Besides DNA origami's chemical composition and architecture, all toxicity studies should consider DNA origami's properties in the context of linkers, carriers, conjugates, and excipients present in their formulation.

### 3.8 Potential for genotoxicity and reproductive effects

An understanding of genotoxicity and reproductive effects is among the safety profile of any new drug entity. It is currently thought that DNA antisense oligonucleotide therapeutics, especially those based on the natural, unmodified phosphodiester backbone, are not genotoxic (Berman et al., 2016). However, due to a theoretical risk for DNA oligonucleotides and their metabolites to cause mutations in the host's genomic DNA via incorporation, chain termination, or triple‐helix‐forming mechanisms, genotoxicity assessment using currently available models is recommended (Berman et al., 2016). A greater risk is expected for therapeutic DNA oligonucleotides containing chemical modifications or crosslinking (Berman et al., 2016). To our knowledge, comprehensive genotoxicity studies of DNA origami are not yet available. Therefore, we hypothesize that the current knowledge, regulations, and models for assessing the genotoxicity of DNA oligonucleotides apply to DNA origami and that differences may exist in their toxicity profiles and be unique to DNA origami due to their complex macromolecular structure, architecture, and stability in biological matrices and tissues.

The information about therapeutic DNA oligonucleotides' reproductive and developmental toxicology is limited. However, a review of current data leads to consensus recommendations for such studies based on the individual product attributes and the species employed to conduct the analysis (Cavagnaro et al., 2014). Of interest, changes in fertility and embryonal and fetal development were more common among DNA oligonucleotide products containing CpG motifs known to activate Toll‐like receptor 9 (TLR9) mediated innate immune responses (Cavagnaro et al., 2014). This information may inform reproductive and developmental toxicology studies of DNA origami.

## 4 IMMUNOLOGICAL CONSIDERATIONS

The immunotoxicity commonly observed in preclinical and clinical studies of traditional TNAs includes fever and fever‐like reactions due to the induction of cytokines and interferons, hypersensitivity due to complement activation, inhibition of plasma coagulation due to interaction with coagulation factors, and hematological changes due to interaction with blood cells and bone marrow. Detailed analysis of these toxicities and the ways in which they relate to TNAs with different compositions, chemical modifications, sequences, and other physicochemical attributes have been discussed elsewhere (Dobrovolskaia & McNeil, 2015a; Levin, 1999). Below, we will recap those related to the DNA oligonucleotides.

### 4.1 Immunotoxicity due to the DNA component

Studies with antisense DNA oligonucleotides of various generations—encompassing those with the phosphodiester (original) backbone, phosphorothioate (containing a sulfur substitution in the phosphate group), morpholino (containing a morpholine ring instead of deoxyribose), chemical alterations (e.g., 2′‐O‐methyl‐, 2′‐O‐methoxyethoxy‐, iodine‐, and bromide‐based modifications), locked nucleic acids, and peptide nucleic acids—created a foundation of the current understanding of the immune recognition of DNA‐based TNAs (Farman & Kornbrust, 2003; Henry et al., 2001). DNA oligonucleotides' sequence, composition, length, type of backbone linkage, presence of CpG motifs, dose and dose regimen, concentration in plasma, and clearance rate were shown to influence their ability to activate immune cells (Henry Giclas, et al., 1997; Henry, Zuckerman, 1997, Henry et al., 2000; Henry, Monteith, & Levin, 1997; Yu et al., 1996, 2000; Zanardi et al., 2012). Toxicities dependent on and independent of hybridization and sequence have also been described (Levin, 1999).

Decreases in leukocytes, neutrophils, and platelet counts were among the most common cellular toxicities observed in clinical trials of DNA oligonucleotide therapeutics (Chi et al., 2005; Holmlund, Monia, Kwoh, & Dorr, 1999). Myeloid cells of the MPS as well as B‐lymphocytes were the primary responders to the DNA oligonucleotide therapeutics (Monteith et al., 1997). Not surprisingly, splenomegaly (Henry, Grillone, Orr, Bruner, & Kornbrust, 1997; Henry, Taylor, Midgley, Levin, & Kornbrust, 1997), lymphoid hyperplasia, reticuloendothelial hyperplasia, and Kupffer cell hyperplasia were observed (Henry, Grillone, et al., 1997; Henry, Templin, Gillett, Rojko, & Levin, 1999; Monteith et al., 1998). Interestingly, extramedullary hematopoiesis in the spleen was noticed in rodents during preclinical studies of antisense oligonucleotides as a compensatory mechanism to erythrocyte loss (Burel et al., 2013; Henry et al., 1999; Henry, Grillone, et al., 1997; Henry, Taylor, et al., 1997; Holmlund et al., 1999; Monteith et al., 1998; Sparwasser et al., 1999; Yacyshyn et al., 1998). Mononuclear cell infiltrates in multiple organs (lungs, kidney, liver) were the predominant cellular toxicity observed in rodents, but not in nonhuman primates and human patients (Burel et al., 2013; Henry et al., 1999; Henry, Grillone, et al., 1997; Henry, Taylor, et al., 1997; Monteith et al., 1998; Sparwasser et al., 1999; Yacyshyn et al., 1998).

DNA oligonucleotides' inhibition of plasma coagulation time was attributed to their polyanionic nature and, in certain cases, occurred through the selective inhibition of the intrinsic tenase complex composed of coagulation factors IXa_B_ and VIIIa, calcium, and phospholipids (Sheehan & Lan, 1998). Activation of the complement system is another type of hematological toxicity commonly reported for DNA oligonucleotides; it occurs through both classical and alternative pathways (Henry, Giclas, et al., 1997; Shaw et al., 1997). The fever and fever‐like reactions due to the cytokine response commonly reported in human clinical trials of antisense DNA oligonucleotides (Advani et al., 2005; Chen et al., 2000; Chi et al., 2005; Holmlund et al., 1999; Monteith & Levin, 1999; Nemunaitis et al., 1999; Rudin et al., 2001; Waters et al., 2000) were attributed to the oligos' recognition by TLR9 (Senn, Burel, & Henry, 2005). Surprisingly, some DNA oligonucleotides, when used at high concentrations, were able to stimulate a cytokine response independent of TLR9 (Senn et al., 2005). This stimulation was attributed to the oligonucleotide recognition by two cytosolic pattern recognition receptors for nucleic acids, IFNβ promoter stimulator‐1 (IPS‐1) and melanoma differentiation‐associated protein‐5 (MDA5) (Burel et al., 2012). Other, nonendosomal pattern recognition receptors of nucleic acids also exist and could potentially contribute to the cytokine response to DNA‐based therapeutics. Therefore, it is generally accepted that, despite the reduction in cytokine response, simply avoiding or inhibiting the interaction between DNA oligonucleotides and endosomal TLRs is insufficient to prevent cytokine‐mediated toxicities, especially when DNA oligonucleotides are used at high doses (Dobrovolskaia & McNeil, 2015a).

It would be logical to expect that many of the findings described in studies of DNA oligonucleotides may also be observed with DNA origami particles. Indeed, some studies confirmed this theoretical expectation. For example, tube‐shaped DNA origami were reported to induce IL‐6 and IL‐12 cytokines via the TLR9‐dependent pathway (Schuller et al., 2011). The lessons learned with traditional DNA therapeutics, therefore, could be used as a guide for better understanding of immunological behavior of DNA origami (Table 3). However, it would be inaccurate to expect the immune recognition of all DNA origami to mimic that of DNA oligonucleotides with similar composition. The unique geometric shapes and structures of macromolecular DNA created by the origami technology may have unique immunological recognitions both via TLRs and cytosolic sensors such as cGAS‐STING. Recent studies with other high‐molecular‐weight TNA created a precedent of such unique properties. Unlike traditional TNA, the type I interferon response by NANPs and sgRNA requires the delivery of these constructs into the immune cells. “Naked” NANPs and sgRNA are invisible to the blood cells, likely due to their higher negative charge preventing their efficient interaction with anionic cellular membranes (Hong et al., 2018; Schubert, Cedrone, Neun, Behlke, & Dobrovolskaia, 2018). Moreover, the immune recognition of NANPs made of double‐stranded RNA was theoretically expected to induce interferon response via TLR3, a pattern recognition receptor for double‐stranded RNA; however, RNA NANPs activated human blood cells via TLR7, which is commonly known as a sensor of single‐stranded RNA (Hong et al., 2018, 2019). Likewise, despite the expectation that DNA NANPs would be recognized by TLR9, the interferon response to these materials also involved the TLR7 pathway (Hong et al., 2018, 2019). Moreover, RNA and DNA nanoparticles were recently shown to activate the RIG‐I pathway in microglial cells (Johnson et al., 2020).

**TABLE 3:** Immunotoxicity of DNA‐based therapeutics

Type of immunotoxicity or activation of PRR	DNA oligonucleotide therapeutics	DNA origami
Decrease in cell counts of leukocytes, neutrophils and platelets	Berman et al. (2016) and Cavagnaro et al. (2014)	TBI
Splenomegaly	Henry, Giclas, et al. (1997); Henry et al. (2000)	TBI
Lymphoid and RES hyperplasia	Henry, Zuckerman, et al. (1997); Henry et al. (2000) and Yu et al. (1996)	TBI
Erythrocyte loss and extramedullary hematopoiesis	Cavagnaro et al. (2014), Henry, Giclas, et al. (1997); Henry, Zuckerman, et al. (1997); Henry et al. (2000), Yu et al. (1996, 2000), Zanardi et al. (2012), Chi et al. (2005)	TBI
Mononuclear cell infiltrates in multiple organs in rodents but not in NHPs and humans	Henry, Giclas, et al. (1997), Henry, Zuckerman, et al. (1997), Henry et al. (2000), Yu et al. (1996, 2000), Zanardi et al. (2012), Chi et al. (2005)	TBI
Inhibition of plasma coagulation	Holmlund et al. (1999)	TBI
Complement activation	Kishi et al. (1990) and Monteith et al. (1997)	TBI
Fever and fever‐like reactions	Berman et al. (2016), Cavagnaro et al. (2014), Henry, Grillone, et al. (1997), Henry, Taylor, et al. (1997), Henry et al. (1999), Monteith et al. (1998), Burel et al. (2013), Sparwasser et al. (1999)	TBI
Activation of TLR9 pathway	Yacyshyn et al. (1998)	Schuller et al. (2011)
Activation of cytosolic PRR (IPS‐1, MDA‐5)	Sheehan and Lan (1998)	TBI

*Note*: Known immunotoxicity of traditional DNA therapeutics is contrasted to that of DNA origami to highlight current knowledge gaps in the field. Studies describing toxicity are highlighted by the references; current knowledge gaps are shown as to be investigated (TBI).

Abbreviations: IPS‐1, IFNβ promoter stimulator‐1; MDA‐5, melanoma differentiation‐associated protein‐5; NHP, nonhuman primates; PRR, pattern recognition receptors; RES, reticuloendothelial system.

Although mechanistic studies of the immune recognition of DNA origami are limited, the available data reveal a difference in the immunological signature of tube‐shaped DNA origami. Interestingly, IL‐1 and IFNα, common biomarkers of the nucleic‐acid‐mediated inflammatory response, were not observed with these materials despite their ability to induce other biomarkers (IL‐6 and IL‐12) (Schuller et al., 2011). It would be interesting to understand the roles of various DNA‐sensing pattern recognition receptors in DNA origami recognition. Such studies may help to explain the differences in the immunostimulatory profile mentioned above (Schuller et al., 2011).

One of the most noteworthy properties of the healthy immune system is its ability to detect and eliminate damaged self‐ and foreign antigens while remaining completely silent toward normal self‐antigens. Such immunologic silence or tolerance is developed during the formation of each host's immune system and maintained via several mechanisms, including elimination, inhibition, and suppression of lymphocytes expressing receptors to self‐antigens.

Alterations in the induction and maintenance of immunological tolerance lead to the immune system attacking the host's own cells and tissues and may result in a variety of autoimmune disorders. Indeed, self‐reactivity to host DNA is a hallmark of the autoimmune disorder systemic lupus erythematosus (Pisetsky, 2000). In this context, one of the key immunological safety questions regarding DNA origami is whether the origami can break the host's tolerance to its own DNA.

While repeated administration of the antigen‐loaded tetrahedron DNA origami was shown to benefit vaccine efficacy, it failed to induce anti‐DNA antibodies (Liu, 2012; Liu et al., 2012). Although these results are encouraging, more research is necessary to understand DNA origami's potential to induce autoimmunity. Such research is especially needed when DNA origami carries functional oligonucleotides, aptamers, and antibodies and is formulated using nonimmunologically inert carriers or excipients, such as liposomes, Polysorbate 80, and polyethoxylated castor oil, because, collectively, these components may skew the immune response toward self‐antigens. DNA origami's effects on existing autoimmune conditions during both the early and the advanced stages of the disease also need to be investigated. Furthermore, the potential contribution of molecular weight, size, method of synthesis, chemical modifications, architecture, and geometric shape need to be understood.

### 4.2 Delivery methods and immunotoxicity challenges associated with them

Traditional TNAs are commonly administered via intravenous and subcutaneous routes, which allows them to rapidly distribute from the injection site to the systemic circulation (Christensen et al., 2013, 2014; Cossum et al., 1993, 1994) and/or reach target tissues via lymphatics (Murakami et al., 2015). Due to the difference in stability between DNA and RNA oligonucleotides, a variety of methods has been explored to increase the stability, improve the circulation time, and ensure the delivery of these therapeutics to target tissues, as outlined above, and as would be required for systemic delivery of structured DNA assemblies to circumvent unwanted immunostimulation or toxicity, or degradation and clearance (Christensen et al., 2013, 2014; Cossum et al., 1993, 1994). Polymer‐ and lipid‐based nanocarriers are among the most extensively explored delivery vehicles for TNAs (Dar, Gopal, & Rao, 2015; Elbakry et al., 2009; Gupta et al., 2015; Halman et al., 2020; Hobel & Aigner, 2013; Kim et al., 2013; Rudzinski & Aminabhavi, 2010; Tanaka et al., 2010; Tsutsumi, Hirayama, Uekama, & Arima, 2008). However, research and clinical experience with these carriers has uncovered several challenges. For example, since complexation between a carrier and TNA is based on electrostatic interaction, the drug (TNA) may release prematurely in plasma before reaching the target site, whereas free carrier with available cationic groups becomes toxic to blood and endothelial cells and may result in hemolysis and coagulation side effects (Boraschi, Costantino, & Italiani, 2012; Dobrovolskaia & McNeil, 2013; Frohlich, 2012; Pantic, 2011). Moreover, some nanocarriers (e.g., those containing protamine, and cationic lipids (DOTAP and CLinDMA)) were found to contribute to the immunostimulatory toxicity inherent to TNAs (Abrams et al., 2010; Kim, Choung, Lee, Kim, & Choi, 2007; Li et al., 1999; Vasievich, Chen, & Huang, 2011). Liposomes and lipoplexes turned out to be even more immunostimulatory and immunotoxic delivery platforms than adenoviral vectors (Sakurai, Kawabata, Sakurai, Nakagawa, & Mizuguchi, 2008). Although nanocarriers helped TNA to overcome certain PK‐related challenges and general toxicity issues, their immunotoxicity still necessitates new strategies. Some of these considerations and approaches have been discussed elsewhere (Dobrovolskaia & McNeil, 2015b) and apply to the new generation of TNAs (e.g., CRISPR/Cas9 and mRNA) as well as NANPs and structured DNA assemblies made using DNA origami. As such, when a polymeric or lipid carrier is considered for the delivery of DNA origami, consideration must also be given to the carrier's PK and safety profiles, as they may change biodistribution and contribute to the toxicity of the DNA origami component of such formulations (Dobrovolskaia & McNeil, 2015a, 2015b).

The use of certain nanocarriers (e.g., PEGylated liposomes) is commonly associated with a high risk of infusion reactions (Szebeni, Simberg, Gonzalez‐Fernandez, Barenholz, & Dobrovolskaia, 2018). Interestingly, the infusion reactions to some PEGylated products were attributed to the anti‐PEG antibodies (Le, Toyofuku, & Scott, 2017; Li et al., 2018; Moreno et al., 2019; Poppenborg et al., 2016; Szebeni et al., 2018; White et al., 2016; Zhang, Sun, Liu, & Jiang, 2016). Questions about the source of these antibodies in a healthy host and their contribution to the immunotoxicity of PEGylated drug products intensified discussions among nanomedicine researchers (Le et al., 2017; Li et al., 2018; Moreno et al., 2019; Neun, Barenholz, Szebeni, & Dobrovolskaia, 2018; Poppenborg et al., 2016; White et al., 2016; Zhang et al., 2016). Therefore, if PEG is present in a DNA origami structure, a carrier, or an excipient present in the origami formulation, the induction of anti‐PEG antibodies and their potential contribution to the distribution, clearance, and toxicity of the DNA origami will need to be investigated. The formation of PEG‐specific antibodies to PEGylated liposomes was demonstrated in several studies and, in some cases, attributed to the presence of the certain innate‐immunity‐modulating impurities, such as endotoxin (Ando et al., 2018; Hashimoto, Shimizu, Abu Lila, Ishida, & Kiwada, 2015; Hsieh et al., 2018; Mima et al., 2017; Shimizu et al., 2015, 2018).

Endotoxins can affect any type of drug product, but they are a grand challenge in the field of nanomedicine; the risk of contamination is greater in formulations with one or more components produced in bacterial cells (Dobrovolskaia, 2015). Therefore, when DNA origami is manufactured using bacterially produced scaffolds, close attention must be paid to the manufacturing process to avoid excessive endotoxin in the final product, as carried out by Shepherd et al. (2019). For example, some cationic and fibrous nanocarriers were shown to exaggerate endotoxin‐mediated inflammation, while being immunologically silent (Dobrovolskaia, 2015).

Based on the current state of the field, we do not predict that DNA origami nanoparticles will exaggerate endotoxin effects. However, such risks may exist when a cationic carrier is used to deliver DNA origami to target tissues. In particular, endotoxins and other microbial components (e.g., CpG DNA, lipoteichoic acid, lipoproteins) may prime immune cells and enhance their response to TNAs by upregulating innate immune receptors that sense foreign nucleic acids. Finally, these contaminants may synergize with immunostimulation caused by carriers, linkers, excipients, and the DNA origami, thereby exacerbating safety risks. Therefore, careful screening and purification from microbial innate‐immunity‐modulating impurities represent important steps in DNA origami's clinical translation path. As mentioned earlier, such data must be considered in the context of carriers and excipients which are present in the formulation and not immunologically inert.

### 4.3 Strategies for reducing undesirable immunostimulation

A general strategy that physicians commonly use to control infusion reactions associated with drug products is premedication with pharmacological agents that affect the activation of the immune cells and biochemical cascade (e.g., dexamethasone, ibuprofen or acetaminophen, diphenhydramine or certidine, ranitidine) (Coelho et al., 2013). Additional measures include a slower infusion rate and administration through alternative routes (Levin, 1999).

In the field of DNA oligonucleotide therapies, chemical alterations to the oligonucleotide backbone and individual monomers have been used to control complement‐activation‐mediated toxicities (Henry, Novotny, Leeds, Auletta, & Kornbrust, 1997; Kandimalla et al., 1997; Webb et al., 2001). For example, phosphorothioate oligonucleotides with 2′‐MOE moiety (Levin, 1999) or methylphosphonate internucleotide linkage (Shaw et al., 1997) oligos with a mixed backbone containing 2′,5′‐ribo‐ and 3′‐5′‐deoxyribonucleotides (Kandimalla et al., 1997; Yu et al., 1996), and chimeric oligonucleotides in which ethyl (cEt)‐modified nucleosides were included on both flanks of the phosphorothioate sequence (Burel et al., 2013) showed reduced complement activation. In addition, a recombinant chimeric protein called the complement activation blocker‐2 (CAB‐2) was also effective in controlling complement‐activation‐mediated toxicities by traditional DNA oligonucleotides (Henry et al., 2002). Cytokine induction by CpG‐containing oligonucleotides was reduced by the use of 5‐methylcytosine and 2′‐MOE backbone modification (Braasch, Liu, & Corey, 2002; Henry et al., 2000).

While these same approaches appear applicable to the field of therapeutic DNA origami, more research is necessary to fully understand their implications, risks, benefits, and unique aspects in the context of complex DNA origami structures. Since high‐molecular‐weight TNAs (NANPs and sgRNA) are invisible to immune cells unless delivered into them (Hong et al., 2018; Schubert et al., 2018), one way to avoid potential immunostimulation is to restrict these materials' delivery to target cells. Additional tactics for limiting the immunomodulatory activity of high‐molecular‐weight TNAs only to that desired for the given indication include the so‐called “smart design approach,” which allows the inclusion of molecular tools such as NFkB decoy oligonucleotides, aptamers, and complement inhibitors, and the “molecular alphabet approach,” which allows tuning high‐molecular‐weight TNA's physicochemical properties (3D structure, shape, sequence, size, etc.) (Chandler & Afonin, 2019).

## 5 CONCLUSIONS

Intrinsic limitations and costs associated with viral delivery and vaccine vectors together with an immense unmet need for new in vivo gene and cancer therapeutics render nonviral vectors such as structured DNA assemblies attractive to investigate for their unique properties with respect to preexisting delivery tools. Specifically, here we highlight the precise, 3D virus‐like geometry of DNA assemblies fabricated using scaffolded DNA origami, offering the ability to control their size on the 10–100 nm scale, as well as the spatial organization and copy number of targeting ligands that may include peptides, sugars, aptamers, lipids, and any other molecular agent, for controlled avidity and specificity in cellular targeting, uptake, and trafficking via biomolecular recognition. In addition, their versatility in gene therapeutic loading that can include siRNAs, ASOs, mRNAs, and CRISPR RNPs with HDR template incorporated render these assemblies of great interest to further investigate in preclinical studies toward possible clinical translation. While many aspects of design, fabrication, and manufacturing at scales required for preclinical and even clinical applications are now solved, numerous questions remain surrounding in vivo biodistribution, stability, tissue/organ/tumor and cell targeting, immune cell stimulation, toxicity, and clearance. Notwithstanding, because the field of structured DNA (and RNA) assemblies has now moved past preexisting design and fabrication challenges, we anticipate a highly productive decade of preclinical research forthcoming with the clinical translation of these novel delivery and vaccine agents for immunooncology and other applications of central importance to human health.

## AUTHOR CONTRIBUTIONS

**Marina Dobrovolskaia:** Writing‐original draft; writing‐review and editing. **Mark Bathe:** Writing original draft; writing‐review and editing.

## CONFLICT OF INTEREST

The authors have declared no conflicts of interest for this article.

## RELATED WIREs ARTICLES

Bioengineered nanoparticles for siRNA delivery
